# Wastewater Treatment by Advanced Oxidation Process and Their Worldwide Research Trends

**DOI:** 10.3390/ijerph17010170

**Published:** 2019-12-25

**Authors:** José Antonio Garrido-Cardenas, Belén Esteban-García, Ana Agüera, José Antonio Sánchez-Pérez, Francisco Manzano-Agugliaro

**Affiliations:** 1 Department of Biology and Geology, University of Almeria, 04120 Almeria, Spain; 2 Solar Energy Research Centre (CIESOL), Joint Centre University of Almería-CIEMAT, 04120 Almería, Spain; abgarcia@ual.es (B.E.-G.); aaguera@ual.es (A.A.); jsanchez@ual.es (J.A.S.-P.); 3 Department of Chemical Engineering, University of Almería, 04120 Almería, Spain; 4 Department of Engineering, ceiA3, University of Almeria, 04120 Almeria, Spain; fmanzano@ual.es

**Keywords:** reclaimed water, advanced oxidation process, microorganisms, concern emergent contaminant, worldwide

## Abstract

*Background*: Water is a scarce resource and is considered a fundamental pillar of sustainable development. The modern development of society requires more and more drinking water. For this cleaner wastewater, treatments are key factors. Among those that exist, advanced oxidation processes are being researched as one of the sustainable solutions. The main objective of this manuscript is to show the scientific advances in this field. *Methods*: In this paper, a systematic analysis of all the existing scientific works was carried out to verify the evolution of this line of research. *Results*: It was observed that the three main countries researching this field are China, Spain, and the USA. Regarding the scientific collaboration between countries, three clusters were detected—one of Spain, one of China and the USA, and one of Italy and France. The publications are grouped around three types of water: industrial, urban, and drinking. Regarding the research, 15 clusters identified from the keywords analyzed the advanced oxidation process (alone or combined with biological oxidation) with the type of wastewater and the target pollutant, removal of which is intended. Finally, the most important scientific communities or clusters detected in terms of the number of published articles were those related to the elimination of pollutants of biological origin, such as bacteria, and of industrial nature, such as pesticides or pharmaceutical products.

## 1. Introduction

Water is the core of sustainable development, but it is a limited resource. The world population growth and climate change have given rise to an alarming decline of freshwater resources and their availability, thus posing a major challenge worldwide. In the last 35 years, the frequency and the intensity of droughts have drastically increased due to the effect of global warming. The number of people and areas affected by water scarcity increased almost 20% in summer of 2017 in Europe [1]. This trend is expected to continue, causing concern across the European Union (EU) and neighboring countries and giving place to important environmental and economic consequences.

Excessive water extraction for agricultural irrigation and for industrial applications [2] is one of the chief menaces to the aquatic ecosystems in the EU, while provision of healthy water is a critical condition for development of economic sectors that depend on water. In response to this problem, hydric resources should be managed more efficiently. A feasible alternative is to apply environmentally sustainable treatments to wastewater to recover them for future purposes. Water reuse is a process with few adverse environmental impacts when compared with desalination or water transfers and offers economic and social benefits. Nowadays, even though water reuse could never solve by itself water scarcity issues, it can help to improve the quality and quantity of the planet’s water supplies.

Although usefulness of treated water is a recognized practice in some EU countries with hydric stress (Greece, Malta, Italy, Cyprus, Portugal, and Spain), only a small fraction of this recycled effluent is reused in them. Additionally, the lack of harmonization regarding permit uses, monitored parameters, and limiting values increases environmental and health risks, which are the main obstacles in carrying out these practices. This non-agreement has caused each member state (MS) to adopt its own guidelines for different water reuse purposes [3].

The World Health Organization (WHO) and the Directive 2015/1787 that amends Directive 98/83/EC on the quality of water intended for human consumption recommend a risk management framework with the aim of developing an approach with minimum quality requirements not only for aquifer recharge and agricultural irrigation but also for drinking, recreational, and recycled water [4]. Countries such as Australia developed their own guidelines for water recycling (NHMRC-NRMMC, 2006). The “Australian Guidelines for Water Recycling” provide a common framework for management of reclaimed water quality and uses, including aquifer recharge and agricultural irrigation. In 2012, the United States Environmental Protection Agency (USEPA) included a wide range of reuse applications [5] and improved these roles. Three years later, in 2015, the International Organization for Standardization (ISO) issued the “Guidelines for treated wastewater use for irrigation projects” [6], including agricultural uses [7]. These roles incorporate limit values of parameters that ensure environmental and health safety of water reuse in irrigation. Spain also has its own regulation. The Royal Decree 1620/2007, established by the Spanish government (National Water Council, Spain’s communities, and local authorities) on 7 December 2007, manages the reuse of reclaimed water in this country. This Royal Decree overturns all other regulations included in articles 272 and 273 of Public Water Resources Domain Regulations. This decree establishes that the water analyses must be carried out in laboratories, which have a quality control system in accordance with general requirements for the competence of testing and calibration laboratories (UNE- ISO/IEC 17025). The microorganisms monitored are intestinal nematodes, *Escherichia coli*, and *Legionella spp.* The main physical-chemical characteristics controlled are turbidity, suspended solids, nitrates, phosphorus, nitrogen, and dangerous substances such as heavy metals.

However, the situation has changed since 12 February 2019 with a European Parliament legislative resolution of the European Parliament and of the Council regarding the proposal for a regulation on minimum requirements for treated water reuse. This regulation sets EU-wide standards that reclaimed water would need to meet in order to be used for agricultural irrigation. In this report are national regulations on water reuse already published by some EU countries as well as a risk analysis carried out by MS for guaranteeing safe use of the treated water. In order to reuse reclaimed water in crop irrigation, different physical-chemical and microbiological steps have been proposed depending on type of crops. Requirements differ according to four water quality classes defined based on the type of crop and irrigation practice and include microbiological parameters (presence of pathogen organisms: *Escherichia coli*, *Legionella spp.*, and intestinal nematodes) and physico-chemical variables [turbidity, biochemical oxygen demand (BOD5), and total suspended solids (TSS)]. In addition, member states should implement programs to monitor environmental matrices in order to establish the impact of reclaimed water on ecosystems, soils, and crops and to assess health risks. For agricultural irrigation, the monitoring programs of the environmental matrices are described in the ISO guidelines (ISO 16075, 2015). These samplings should be carried out taking into account the minimum requirements concerning the frequency of testing in order to establish a risk management plan. In this way, the potential additional hazards may be addressed.

The choice of the best treatment for reclaiming wastewater with the aforementioned requirements depends on its later purpose. Consideration should be given to adding the chemical procedures applied and the residual products resulting from the treatment. This prevents contamination and salting problems from affecting freshwater sources. Therefore, lower cost, robust, and more effective processes to decontaminate and disinfect wastewater are required without endangering human health or stressing the environment by the treatment itself, mainly in sub-developed countries. In this context, advanced oxidation processes (AOPs) are considered a highly competitive technology regarding water treatments for the removal of organic pollutants classified as bio-recalcitrant and for the inactivation of pathogen microorganisms not treatable by conventional techniques. AOPs were first suggested for drinking water treatments in 1980 (NHMRC-NRMMC, 2011). In later years, they were widely studied as oxidizing treatments applied to different wastewaters. AOPs are defined as the oxidation processes related to the generation of reactive oxygen species (ROS) such as hydroxyl radicals (HO) in enough quantity to produce reclaimed effluents. HO· radicals have high redox potential (2.8 eV) and are non-selective [8]. They are capable of attacking organic compounds through four pathways: hydrogen abstraction, combination or addition of radicals, and electron transfer [9]. Their reaction with organic contaminants generates carbon radicals (R· or R·−HO), which may be transformed to organic peroxyl radicals (ROO) with O_2._ All the radicals further react accompanied by the formation of other reactive species such as super oxide (O_2_·−) and hydrogen peroxide, leading to chemical destruction and, in certain cases, the mineralization of water target pollutants. When an AOP is applied as a tertiary treatment, HO· radicals are generated in situ due to their short lifetime by different procedures, including a mixture of oxidizing agents (ozone and hydrogen peroxide), ultrasound (US) or irradiation (UV), and catalysts [10]. The most frequent catalyst is titanium dioxide (TiO_2_). When the TiO_2_ particles are illuminated by UV light, they are excited and generate valence band holes where HO· are produced in contact with water [11]. However, the recycling and the recovery of these suspended TiO_2_ particles become cumbersome and expensive, making the use of suspended systems not viable. As an alternative, new systems have been developed using this immobilized catalyst [12,13]. However, the combination of hydrogen peroxide with Ultraviolet-C light (UVC) radiation results in a most effective procedure for yielding HO· [14]. On the other hand, the use of iron species as free catalysts for producing HO in Fenton processes has widely been studied, but its application on wastewater such as tertiary treatments in real conditions is restricted since the optimal pH is 2.8. For this reason, studies have proposed three modified Fenton process: heterogeneous Fenton, photo-Fenton, and electro-Fenton [15]. In order to carry out the photo-Fenton reaction, the traditional Fenton system is exposed to the UV light with the aim of improving the photo-reduction of dissolved ferric iron (Fe^3+^) to ferrous iron species (Fe^2+^). In the electro-Fenton reaction, the two Fenton reagents can be produced with electrochemical procedures [16]. Another HO· and hydrogen peroxide generation system is the water sonolysis [17]. This treatment is less studied in wastewater because the initial operational costs are very high, and the treated volumes are very small in comparison with other methods. Not only are HO· oxidant agents, but they are also sulphate radicals. These species are highly reactive, and they have a short life cycle. Therefore, the HO can be generated from them at alkaline conditions.

As has been described previously, HO· radicals, generated during the application of AOPs on secondary effluents are able to remove wastewater toxic products and transform them to non-dangerous pollutants, providing a solution for wastewater treatment [10,18]. Besides oxidation by HO, other simultaneous reactions can take place during the treatments with AOP, giving rise to destruction of target compounds in wastewater. The function of these non-radical oxidative mechanisms in the pollutant removal may be insignificant or dominant depending on the reaction conditions and the applied AOP type.

In recent years, small concentrations of inorganic, organic, and mineral compounds in the aquatic environment have increased noticeably, mainly by human activities such as excessive and rapid industrialization, urban encroachment, and improved agricultural operations. One feasible option for eliminating organic pollutants from wastewater is the application of AOPs or their combinations with other treatments. These methods have been commonly recognized as being highly capable for removing recalcitrant contaminants or being used as pretreatment to transform contaminants into shorter-chain compounds that can be treated after by traditional biological processes. One AOP or a combination must be appropriately selected for remediation of a specific industrial or urban wastewater, considering factors such as wastewater characteristics, technical applicability, regulatory requirements, economical aspects, and long-term environmental impacts.

AOPs such as photocatalysis and photo-Fenton have been proposed as tertiary treatments for urban effluents due to their ability to detoxify wastewater streams containing persistent contaminants. The treatment of industrial wastewater (IWW) effluents is a very complex challenge due to the broad array of substances and high concentrations that it can contain. Treatment by activated sludge is more efficient and less expensive for removing high concentrations of organic compounds. However, there are some circumstances where AOPs may offer some advantages. AOPs typically have a small footprint and can be easily integrated with other treatment processes. They could be used to remove non-biodegradable substances that persist after biological treatment. In fact, some IWWs are toxic or bio-recalcitrant to activated sludge treatment due to the high dissolved organic carbon (DOC) concentration. It was proven that AOPs can be used to partially degrade toxic compounds for obtaining effluents with more biodegradables prior to the biological process [19]. In this way, studies about combined AOPs and biological technologies for treating some complex IWWs have greatly increased in recent years [18,20,21]. For that, the reuse of IWW as a harmless hydric resource under adequate sanitary conditions is a real possibility. According to this, novel treatments based on AOPs and their combination with conventional treatments are being evaluated, covering an extensive range of IWWs generated from different processing industries. Olive oil production is one of the major agronomic activities in the Mediterranean region [22]. However, the high phenolic toxicity of resulting effluents generated serious environmental issues in these places, making it necessary to find a suitable treatment in order to diminish the environmental impact of their discharge. A possible solution is to apply a treatment with active sludge as pre-treatment to enhance the biodegradability of IWW [23], since processes such as electrochemical oxidation [24], Fenton oxidation [25,26], and ozonation [27,28] can only reach partial decontamination even after extended times. Another industry that obtains large amounts of IWW is the winery industry, as Europe is the main producer of this drink. In scientific literature, some studies demonstrated high efficiency on organic matter removal by ozone [24,25] or photo-Fenton processes [29,30], pointing out the process combination to improve traditional techniques. If attention is paid to textile industries, they generate a negative environmental impact due to discharge of dyes and chemicals in stream water. In recent years, research has reported that these industrial wastewaters must be treated in the first place by applying a biological system and after with AOP oxidation to complete the treatment of textile IWWs. AOPs such as ozone, UV/H_2_O_2_, TiO_2_-assisted photocatlysis ozonation, or Fenton, photo-Fenton, hydrogen peroxide, and electro-oxidation processes have been evaluated to treat these types of IWW with promising results [18].

In addition to industrial effluents, it is also recognized that municipal wastewater treatment plants (MWWTPs) represent a relevant reserve of environmental water contamination. The total charge of organic pollutants discharged by MWWTPs depends on the number of residents and the pollution received from local industries connected to the urban sanitary system. A wide variety of toxic residues (chemicals or biological products) are generated daily by different sectors, which can be classified as hazardous or toxic due to the possible adverse effects that can generate (neurotoxicity, endocrine disruption, cancer) [31]. Among the contaminants present in WWTP effluents are personal care products, pharmaceuticals, pesticides, gasoline additives, flame retardants, drugs, plasticizers, and a long list of chemicals commonly identified as “contaminants of emerging concern (CECs)” [32,33]. These compounds are found at ng/L–µg/L concentrations in MWWTP effluents, but they are not regulated. WWTPs are considered as the main pathway of entry of CECs to the environment. During the water treatment processes, or once in the natural environment, these compounds can also be transformed by a variety of chemical, photochemical, or biological processes that lead to the formation of transformation products (TPs), which can eventually be more persistent or dangerous than the original compounds [34,35]. The inefficient removal of CECs by MWWTPs is a serious limitation for water reuse in regard to the safety/sustainability of reuse practices such as irrigation in agriculture or gardens and golf courses [36]. Therefore, the regulations do not permit that the biologically treated wastewater can be directly reused because of its content of health hazard micropollutant. These dangerous products can be accumulated in vegetables and soils with great impact on drinking water resources and food security [37]. This situation requires the development of alternative remediation technologies to limit the discharge of these compounds in the environment. Numerous scientific studies were recently reported proving the effective removal of micropollutants contained in actual urban wastewater [38,39]. Membrane bioreactors technology (MBR) combining conventional activated sludge (CAS) treatment with a membrane filtration system was reported as an alternative to increase the effluent quality decreasing the membrane cost [40,41]. On the contrary, MBR is not available operational technology for eliminating micropollutants due to membrane-fouling control. In this way, membrane aeration, permeability loss, and membrane replacements are factors with high operation costs [42]. In this sense (and to replace MBR technology), some authors have proposed the use of solar AOPs as tertiary treatments for CECs removal due to the use of solar energy diminish investments costs [43], resulting in WW treatments that are simple, robust, and inexpensive. Among the AOPs, the more studied is the heterogeneous photocatalysis using TiO_2_ as a catalyst. However, it was demonstrated that it is not effective because long treatment times are necessary for total elimination of microcontaminants [10]. Another AOP that produced low microcontaminant degradation is the solar photo-Fenton working at neutral pH [44]. In order to avoid the iron precipitation in neutral pH conditions, chelating agents are needed for keeping the catalyst in solution. Commonly, the complexing agents exist in the WW but are removed during secondary biological treatment or drinking water treatment phases. In nature, there is a wide range of agents that can be very useful for keeping the dissolved iron in the course of the solar photo-Fenton process and to stabilize the free radical production [45,46,47].

Wastewater effluents contain not only harmful inorganic and organic compounds but also pathogen microorganisms. Chlorination, UV-C radiation, and ozone are treatments traditionally used for microorganisms’ inactivation in WWTPs. WW disinfection mainly focuses on specific groups of bacteria included in the water reuse regulations for different uses, which include total and fecal coliforms, *Crystosporidium* sp., or *Legionella* sp. The addition of chlorine substances (chlorine gas, sodium hypochlorite, or calcium hypochlorite) is the most cost-effective treatment and has proven to be lethal against a wide range of wastewater pathogens microorganisms. Microorganisms’ inactivation is reached by different cellular oxidation mechanisms and inhibition of enzymatic activity together with the damage of the cell membrane [48]. In relation to the WW disinfection under UV-C radiation, it was demonstrated that the photons were absorbed by the microorganisms’ genetic material (DNA), thus avoiding the cellular replication. In addition, the accumulated UV-A energy per unit of treated water volume dose (QUV), in terms of kJ/L, is a key parameter to monitor the microorganism inactivation under UV radiation in the function of treatment time when the system is photo-limited [49]. Nonetheless, the interest in ozone treatments is increasing since this chemical has the power to inactivate microbial cells and to decrease the load of organic chemicals [46,50].

Currently, AOPs are shown as alternative technologies due to their ability to destroy a broad variety of contaminants and to kill microorganisms from WW. However, these treatments are being investigated in order to diminish associated operational costs and ensure the feasibility. The main AOPs studied for water purification are TiO_2_, photocatalysis, UV/O_3_, UV/H_2_O_2_, Fenton, and photo-Fenton [51]. In order to evaluate their feasibility, model microorganisms of fecal contamination are selected due to their great immunity to most water disinfection methods conventionally applied. Among them, the most common are *Escherichia coli* (*E. coli*), *Cryptosporidium* sp., or *Bacillus* sp. These microorganisms are extremely dangerous to human health. *E. coli* can hydrolyze conjugated estrogens by sulfatase and glucuronidase enzyme. Regarding protozoan microorganisms, *Cryptosporidium* is known for its resistance to chlorination processes, causing intestinal infections in the human population [10]. On the other hand, *Bacillus* sp. is a facultative anaerobic bacteria that can live with a low amount of dissolved oxygen. There are many *Bacillus* species existing in nature—some of them have a high wastewater purification ability to decompose highly concentrated organic matter in a short time, and they secrete a large quantity of enzymes that can decompose excess sludge. However, other species are toxic if they appear in the treated water after secondary treatments, and tertiary processes such as chlorination are not capable of inactivating them [52].

The use of UV radiation combined with H_2_O_2_ or TiO_2_ increases the efficiency of the inactivation process. The action mechanism of microorganism inactivation by UV differs from the pathogen inactivation by UV/H_2_O_2_. When both WW disinfection techniques are compared, the treatment times are shorter when the combined process is applied [53]. Another type of UV radiation is UV-C, which has been demonstrated to be more effective than UVA/TiO_2_ or UV/sono-chemical treatments since it has a great disinfectant power, obtaining an inactivation reduction of 6-log in 10 min of treatment time. However, the photo-reactivation of bacteria occurred at 72 h after the end of the applied process [54]. Another effective, solar driven AOP is the photo-Fenton process. When the pH values are increased, the ferrous iron solubility decreases, leading to its precipitation as Fe^3+^ hydroxides. This fact can be an issue when the photo-Fenton process is selected for disinfecting wastewater, thus the survival of the majority of the monitored pathogens decreases or even dies at acidic pH (pH < 3). In order to deal with this blockage and evaluate the inactivation of pathogen organisms by the photo-Fenton process, several studies were performed at pH over 4, where microbes are able to survive [49,55,56]. In order to inactivate microbial cells for photocatalysis by TiO_2_, a critical fact is the internal cellular damage produced by acts of reactive oxygen species (ROS), such as HO·, just as for photocatalysis by TiO_2_. This effect on vital compounds of the cell begins with the photon absorbance through the plasmatic membrane of microorganisms causing lethal physical damage followed by an oxidative attack by hydroxyl radicals on the cellular walls, generating oxidative stress and pores and causing loss of their permeability [55]. This circumstance is dependent on the amount of HO· and the availability of iron along the photocatalytic treatment.

Nonetheless, current microbial pathogen identification methods in WW have reported the presence of a widespread range of other microbes. These organisms are considered “emerging pathogens” with an inherent alarm to the population due to their appearance in reclaimed waters and discharged waters to the environment. An example of emerging pathogens is the antibiotic-resistant bacteria (ARB). The presence of antibiotics in effluents has increased in the past year. In particular, urban [56] and hospital wastewaters [57] are among common ARB spread and anthropogenic sources into the aquatic world. Finding an effective and advanced technology to remove antibiotic compounds from treated water has been a major study focus for many years. Mechanical procedures such as nanofiltration and ultrafiltration after a traditional activated sludge have demonstrated that the removal of antibiotics increased by up to 30% [58], but these techniques do not remove microcontaminants; they only transfer them from one point to another. For this reason, the AOPs can be applied to clean the effluent containing pharmaceutical products as an environmentally friendly process by using reusable catalysts and solar light. TiO_2_ photocatalysis, Fenton, and photo-Fenton processes have emerged as promising wastewater treatment technologies.

Presence of ARBs in the WWTP effluents discharged into aqueous ecosystems or reused for irrigation in agriculture indicates that disinfection routine practices do not successfully control the spread of these pathogen organisms into the environment. ARBs are frequently found in WW effluents from hospitals, MWWTPs, and wastewater from cattle (known as “grey waters”). Currently, the main conventional disinfection methods are chlorination, application of ozone, and UV-C. Regarding chlorine compounds, it is reported in bibliography that ARB inactivation rates are not lower than those of total heterotrophic microorganisms, and even the proportion of numerous ARBs can be raised after adding chlorine compounds [59].

Accordingly, the external and the internal mechanisms of how the chlorination process affords to increase the concentrations of ARB and antibiotic resistant gene (ARG) in WW remains uncertain, hence more studies in this field are mandatory. Concerning UV-C radiation effects, the available information indicates that this treatment is not effective in the death of ARB and the removal of ARG under UV-doses around 30 mJ/cm^2^, which are commonly used. When mJ/cm^2^ is increased, the microorganism inactivation rate is increased too, thus achieving the total log reduction. According to the studies found in literature, inactivation of 4–5 log of cell ARB requires low UV-doses from 10–20 mJ/cm^2^ in comparison with those required for eliminating ARG (UV-doses from 200 to 400 mJ/cm^2^). This indicates that chlorination and UV treatments may not produce an important impact over concentrations of ARB and ARG in WW, although the ways of elimination of these biological products are not clear. On the other hand, the effect of ozonation on ARB has been evaluated in few investigations. They reported that this method is not feasible for killing ARB or eliminating ARG. Nevertheless, other treatments are being considered in order to improve the efficacy of wastewater disinfection and to overcome numerous drawbacks of the aforementioned conventional technologies, decreasing the associated operational expenditures as well. AOPs driven under solar light such as Fenton and photo-Fenton processes have been evaluated on WW for inactivating ARB and ARG. The results extracted from these studies confirmed its lethal power on natural ARB from MWWTPs secondary effluents. Conversely, and depending on the type of resistant gene, its efficacy is lower when the ARG concentrations are analyzed, obtaining total damage in some monitored AG [60,61,62]. The presence and the spread of resistant microorganisms in the effluents from MWWTPs disposed into reused effluents are some of the biggest threats to humanity associated with the domestic use of wastewater. These facts reveal the inefficacy of traditional wastewater treatments and disinfection processes for controlling the spread of pathogenic microorganisms and microbial resistance into the aquatic environments. Nowadays, although some research is being carried out to control the spread of ARB and ARG in aquatic environments, the biological procedures to effectively deactivate these microorganisms remain unclear. These studies pave the way to addressing this challenge.

The objective of this work was to review the state of the art of scientific publications on wastewater and advanced oxidation. In this way, it is possible to establish the true state of research in this topic, defining trends and tracing possible lines of work for the development of future research. To this end, an extensive bibliometric analysis was carried out, and scientific communities were established based on the keywords defined in each article.

## 2. Materials and Methods

Web of Science (WoS) and Scopus are the most important databases of scientific literature. However, there are works comparing them and concluding that using Scopus, the largest database of peer-reviewed scientific articles in the world, is the best option [63]. Studies determine that, while 54% of Scopus titles are indexed in WoS [64], 84% of WoS titles are also indexed in Scopus. Thus, comparative studies conclude that it is much more effective to use Scopus than WoS in bibliometric analysis. Therefore, in bibliography, a large number of bibliometric works using this database can be found [64,65]. Scopus contains almost 40,000 titles belonging to more than 10,000 publishers, and the analysis of these allow the scientific community to identify where, who, when, and how research is taking place in a given scientific area. Therefore, Scopus was the database chosen by us to carry out this analysis.

In the present analysis, a full search of the Elsevier Scopus database was conducted using [TITLE-ABS-KEY (Wastewater and “advanced oxidation”)] as the search query. This resulted in 3208 documents obtained between 1990, the year of first publication, and 2018, the last full year from Scopus database. If the search criteria are modified, the results obtained can be significantly different. Similarly, continuous updates or modifications of the database may also result in certain differences in the result obtained. On the other hand, it must also be taken into account that some of the elements analyzed are the keywords entered by the author or the editor, and these do not always fit the subject matter of the articles. These small anomalies do not invalidate the methodology, and Scopus is considered the best option in bibliometric analysis.

In analyzing the keywords, it was considered that there are terms mentioning the same concept. For example, ozonation and ozonization. Therefore, these keywords were considered as one. On the other hand, keywords that do not contribute to the analysis, such as “article”, were discarded. The items analyzed were: evolution of scientific output, publication distribution by countries and institutions, and keywords. The software tool VOSviewer (http://www.vosviewer.com/) was used in the detection of scientific communities. These scientific communities are represented in graphs, and they are studied as nodes that establish connections between them giving rise to complex networks that determine the relationships within the whole.

## 3. Results and Discussion

### 3.1. Progression of Scientific Output

**Figure 1:**
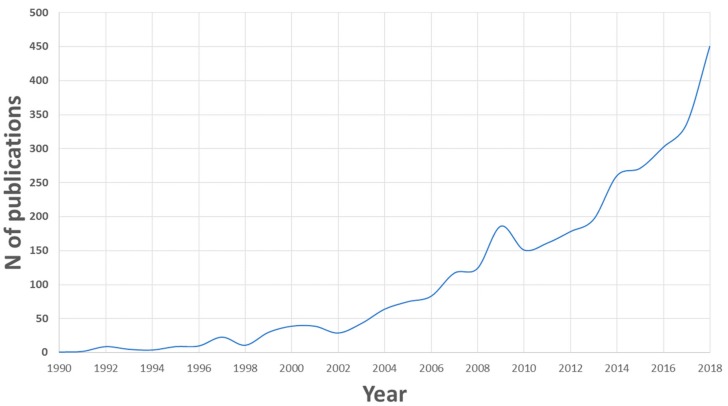
Trend of the number of publications per year in wastewater and advanced oxidation from the years 1990–2018.

The search returned 3208 documents. More than 90% of these documents are written in English, but there are also articles written in other languages, such as Spanish, French, Russian, or Chinese. No document from Scopus was excluded. Figure 1 shows the evolution of the number of documents on wastewater and advanced oxidation since 1990. As can be seen, the topic is of recent interest, because only in the last 30 years has the advanced oxidation in wastewater treatment been used.

There has been continuous growth since the first year analyzed, but this occurs at two speeds. In the period 1990–2002, there is a slow growth in the number of annual publications, while in the period 2002–2018, the growth is much greater with a publication intensity greater than 25 articles per year, reaching the absolute maximum in the year 2018 with 450 articles published.

### 3.2. Publication Distribution by Countries and Institutions

**Figure 2:**
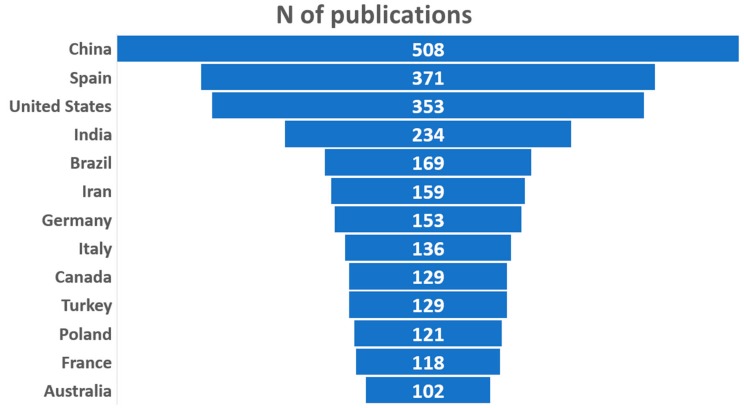
Representation of the countries with the highest number of publications on wastewater and advanced oxidation.

Figure 2 shows, in a funnel chart, the distribution by country of the scientific production on wastewater and advanced oxidation. Thirteen countries have published at least 100 articles on this subject in the period under review. Of these, China stands out with 508 articles. It is followed, in this order, by Spain, the USA, and India, with 200–400 publications. The rest of the countries have published between 100 and 200 documents. China’s water pollution has increased in the last 40 years due to population and industrialization growth. Compared with the European Union and the United States standards, China’s wastewater discharge standards still have shortcomings, and there exist many challenges in this field. For this reason, the Chinese government is putting more effort into exploring new wastewater treatments [66]. In the United States, the potential use of municipal reclaimed water in the power sector has led to an increase in the number of publications about wastewater reclamation treatments. The implementation of technologies capable of supplying reclaimed water for electric power plants is a major challenge for this developed country [67]. As it is presented in Figure 2, Spain holds second place in the ranking with 371 publications related to the studies about advanced oxidation treatments applied to wastewater. In recent years, the changes of legislation at the European level have been a point of departure for increasing the investigation in this area, especially in countries with a huge water scarcity, such as Spain.

**Figure 3:**
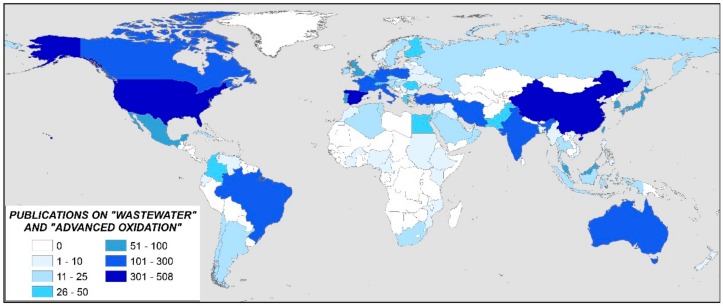
World map representing the scientific production by countries.

A total of 87 countries have published at least one article in this scientific field. However, the 13 most important countries account for almost 85% of all publications. In Figure 3, the countries with at least one publication are represented in a color-coded world map.

**Figure 4:**
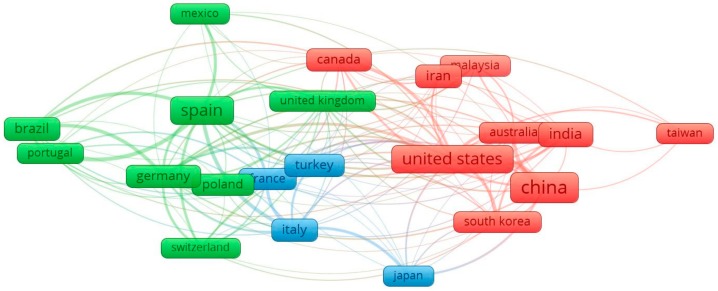
Graph of the analysis of communities by country representing the relations established with other countries.

Figure 4 shows an analysis of the relations established between the different countries based on articles published by several countries. In the analysis of communities, each node represents a country, and from this, there are as many lines of union as there are relations at the level of publications with other countries. The size of each node refers to the number of publications carried out in that country, and the thickness of each union line refers to the number of collaborations carried out with another country. It can be observed that, in the analysis, there are three clusters, each identified with a color. The largest cluster is represented in red and is dominated by China. The USA also plays a very important role in this cluster. The rest of the countries belonging to the cluster are Australia, Canada, India, South Korea, Taiwan, Malaysia, and Iran. The second cluster, represented in green, is formed by European and Latin American countries. The central role is occupied by Spain, and the rest of the countries are the United Kingdom, Switzerland, Poland, Germany, Portugal, Brazil, and Mexico. The third cluster is made up of France, Italy, Turkey, and Japan and is represented in blue.

**Figure 5:**
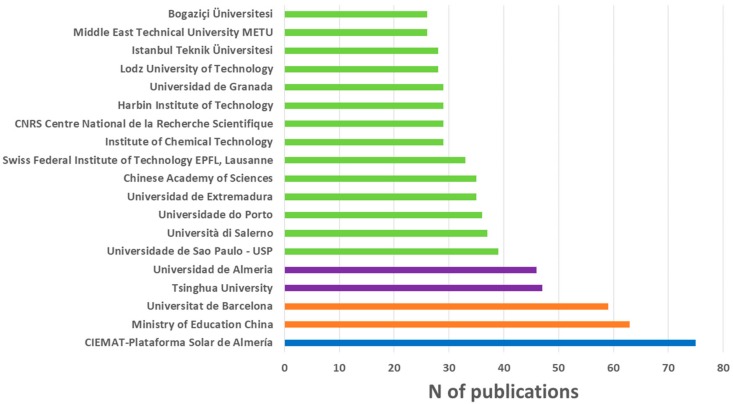
Main institutions related to scientific production in wastewater and advanced oxidation.

Figure 5 shows the institutions with more than 25 publications on wastewater and advanced oxidation. Of these, five are Spanish (CIEMAT-Plataforma Solar de Almería, Universitat de Barcelona, Universidad de Almeria, Universidad de Extremadura, and Universidad de Granada), four are Chinese (Ministry of Education China, Tsinghua University, Chinese Academy of Sciences, and Harbin Institute of Technology), three are Turkish (Istanbul Teknik Üniversitesi, Middle East Technical University METU, and Bogaziçi Üniversitesi), and there is an institution of Brazil (Universidade de Sao Paulo), Italy (Università di Salerno), Portugal (Universidade do Porto), Switzerland (Swiss Federal Institute of Technology EPFL), India (Institute of Chemical Technology), France (CNRS Centre National de la Recherche Scientifique), and Poland (Lodz University of Technology). It is striking that, of the 20 institutions with the highest number of publications, none are American, despite the fact that the USA is the third country, after China and Spain, with the highest number of publications in wastewater and advanced oxidation. This is because, while in other countries such as Switzerland, the majority of research in this field is carried out in a single institution, in the USA, the research is not led by a single center. There are many institutions spread all over the country that publish in this scientific field without highlighting, as far as the number of publications is concerned, any of them.

### 3.3. Keyword Analysis

**Figure 6:**
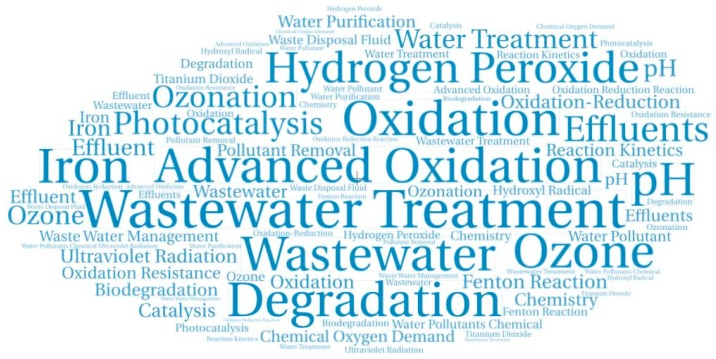
Cloud-word with the more representative keywords.

In order to carry out the analysis of the keywords, the data were previously cured, eliminating irrelevant terms and unifying terms that allude to the same concept. This analysis allowed us to establish the true state of research in a topic, defining trends and tracing possible lines of work for the development of future research. Figure 6 is a cloud-word showing the 32 keywords that appear in more than 300 publications in this area. The size of each keyword represents the relative proportion of each term to the total number of words.

Among the 160 keywords that appear in more than 80 publications, many of them allude to water-related parameters such as pH, temperature, total organic carbon, or organic matter, and many others refer to water pollutant compounds such as phenols, sulfur compounds, aromatic compounds, or antibiotics.

**Figure 7:**
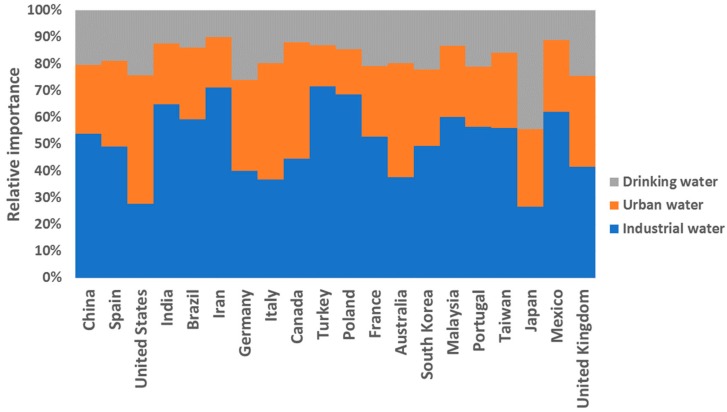
Relative importance given by each country to each type of water.

On the other hand, a large number of articles appear around keywords related to three water types—industrial, urban, and drinking water—with 1357, 786, and 468 articles, respectively. Figure 7 shows the relative importance that each of the 20 most important countries, in terms of the number of publications, attaches to each of these types of water.

It can be observed that, on average, the greatest importance is given to industrial water with 50%, then urban water with 30%, and finally drinking water with approximately 20%. However, if we look in detail at the data for each country, there are countries such as the United States in which the main concern is focused on urban water. Research in Australia also places the greatest importance on urban water. In contrast, urban water has very little relative importance in the research of countries such as Poland, whose research focuses on the study of industrial water. Finally, special consideration should be given to the data obtained in Japan, where almost half of the articles focus on drinking water. This fact is especially important when looking at the year 2011, when the Great East Japan Earthquake occurred, leading to the Japanese Prime Minister declaring the state of nuclear emergency. From that moment on, the Ministry of Health, Labour and Welfare, Japan (MHLW) showed its great concern for the healthiness of tap water and defined a series of indications [68].

**Figure 8:**
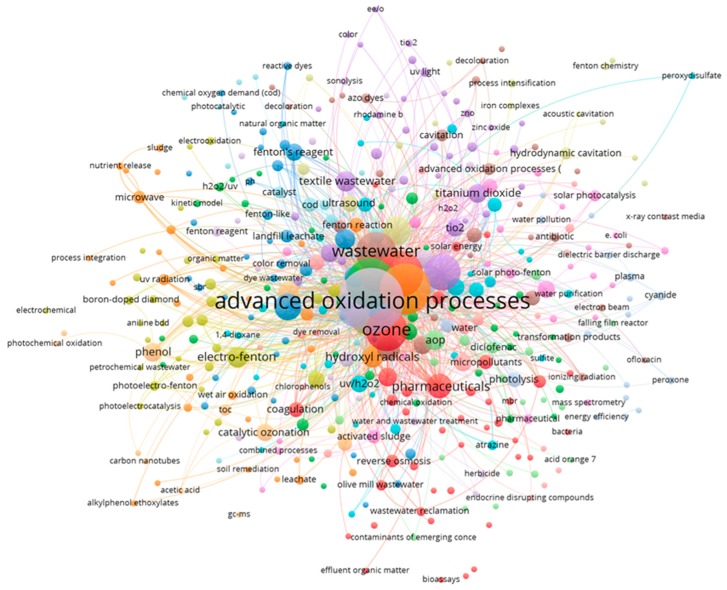
Scientific communities grouped in clusters based on the analysis of keywords in publications on wastewater and advanced oxidation.

The analysis of the keywords by means of the detection of scientific communities allowed us to group the publications in clusters of functions of the keywords used. Figure 8 shows the 15 communities that appeared in the analysis carried out, identifying each cluster with a color. The size of each cluster refers to the importance of the keywords around which the cluster is built, and the thickness of the lines of union between two clusters refers to the number of interactions established between two different communities. In each cluster, there is a variable number of keywords, but it was detected that, in all cases, there are always keywords referring to three aspects related to the analysis we carried out: AOP treatment, wastewater, and targets. Thus, in Table 1, each cluster is identified with the color with which it appears in Figure 8 and with the set of keywords that appear grouped around the three aspects mentioned above.

Table 1 presents 15 clusters identified from the keywords analyzed relating the AOP treatments (alone or combined with biological oxidation) with the type of wastewater and the target pollutant of which removal is intended. From this, several conclusions can be drawn. On the one hand, ozone-based treatments are mainly used for pharmaceutical removal in a wide range of water matrices, from drinking water (cluster 16) to hospital or olive mill wastewater (cluster 1) [69]. Fenton and assisted Fenton processes find application in complex media such as industrial wastewater from different industrial sectors to remove dyes (cluster 4 and cluster 7), COD, and several persistent organic pollutants (cluster 9 and 14). Among the radiation based AOPs, heterogeneous photocatalysis (TiO_2_) has been widely studied for industrial wastewater treatment (cluster 13), although hydrogen peroxide-based treatments such as photo-Fenton and H_2_O_2_/UVC gained attention for municipal wastewater to remove micropollutants (mainly pharmaceuticals) as well as water and wastewater disinfection (cluster 9 and 12).

**Figure 9:**
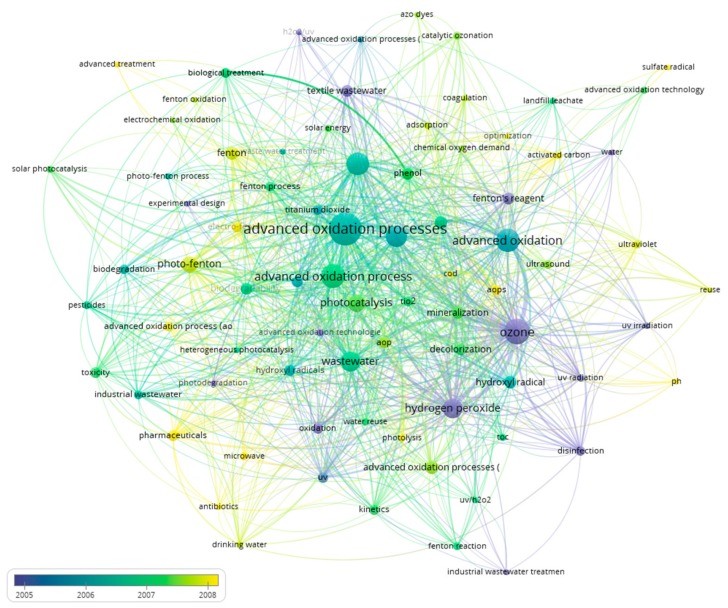
First period of evolution in advanced oxidation for wastewater treatment (2005–2008).

**Figure 10:**
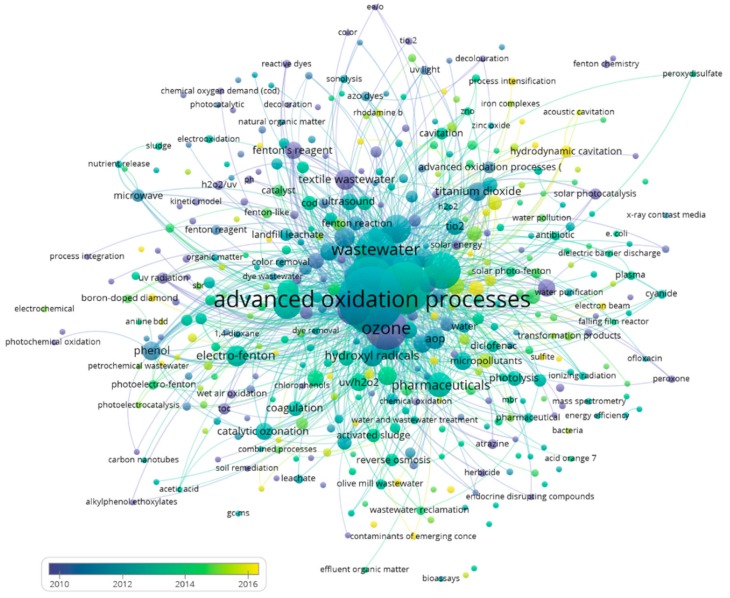
Second period of evolution in advanced oxidation for wastewater treatment (2010–2016).

Finally, if an analysis is carried out on the importance of keywords in time, two periods can be identified. The first period extends from 2005 to 2008 (Figure 9), while the second period extends from 2010 to 2016 (Figure 10).

In the first period, in its beginning toward the year 2005, there were numerous articles that presented keywords such as ozone, hydrogen peroxide, or UV irradiation. Then, mid-term was when advanced oxidation processes, solar energy, or biodegradation stood out. Finally, these lost their leading role to keywords related to the processes of Fenton and microwave, photo-Fenton, drinking water, or pharmaceuticals. This trend was confirmed in the second period, with the Fenton gaining prominence as it progressed at the end of this period, highlighting, above all, solar photo-Fenton.

## 4. Conclusions

Analyzing the number of publications on wastewater and advanced oxidation from 1990 to 2018, a great increase in the evolution is shown, demonstrating the growing scientific interest in this research area. The evolution in the number of publications is especially outstanding from the year 2002 onwards. This growing interest is likely due to the growing need to solve the problem of water purification in all points of the planet. However, the trend regarding the number of publications was not the only item studied. Other variables also received our attention. Thus, it was shown how China and Spain are the countries that publish the largest number of scientific publications in this area, and how five of their institutions (two Chinese and three Spanish) lead the scientific production on wastewater and advanced oxidation, with the CIEMAT-Solar Platform of Almeria leading the ranking. The analysis of the keywords allowed us to establish different scientific communities, focusing each of them on different AOP treatments, type of wastewater, and the target pollutant to remove. The most important scientific communities in terms of the number of published articles are those related to the elimination of pollutants of biological origin, such as bacteria, and of industrial nature, such as pesticides or pharmaceutical products. The treatments mostly present in these articles are those related to ozone/UV, heterogeneous photocatalysis, and H_2_O_2_/UV. The following points are worthy of special attention for future research in the advanced oxidation field:

(1) The current research in wastewater treatments with AOPs is mainly focused at a pilot scale in batch mode. The evaluation in real conditions in continuous flow mode is mandatory for large-scale implementation.
(2) Strengthening the investigation on the removal of contaminants of emergent concern and bacterial inactivation for wastewater reuse using different AOPs.
(3) Contamination caused by antibiotics and ARBs/ARGs should be highlighted as a worldwide health and environmental concern. In-depth research about removal mechanisms of antibiotics and ARBs/ARGs using AOPs is required.

## Figures and Tables

**Table 1:** Summary of different advanced oxidation processes (AOPs) associated with target wastewater and compounds.

Cluster	Color	AOP Treatments	Wastewater	Targets
1	Red	Ozone, ozone/UV, TiO_2_,	Hospital, olive mill, petroleum refinery	Pharmaceuticals, bacteria
2	Dark Green	Electrochemical, H_2_O_2_/UV, heterogeneous photocatalysis, TiO_2_	Textile industrial, paper industrial	Bisphenol a, formaldehydo, lignin, pharmaceuticals, pesticides, antibiotic resistant bacteria, endocrine disruptors
3	Dark Blue	Fenton	Coking, textile, paper industrial, oil refinery, winery	Endocrine disruptors, acetaminophen, colors, COD
4	Dark Yellow	Electro-Fenton, anodic oxidation, sonoelectrochemistry	Industrial textile, petrochemical	Organic pollutants, organic matter, dyes
5	Dark Purple	TiO_2_, Fenton, photo-Fenton, UV	Livestock, winery, textile	Persistent organic pollutants
6	Cyan	Electrolysis, Fenton, ozone, peroxy- and peroxymonodisulfate, UV/persulfate, UV/H_2_O_2_, UV/TiO_2_, zero-valent ion	Saline	COD, 1,4-dioxane, atrazine, dyes
7	Dark Orange	Electro-Fenton, Fenton, microwave, ozonization, UV radiation, wet air oxidation	Dyeing	Phenols, heavy metals
8	Brown	Cavitation, ionizing radiation	Oily	Emerging contaminants, azo dyes
9	Pink	H_2_O_2_, photo-Fenton, UV,	Slaughterhouse, urban	*E. coli*, pharmaceuticals, COD
11	Green	Ozonation, UV/Fenton	Municipal	Pharmaceuticals
12	Blue	H_2_O_2_, UV, UV/H_2_O_2_	Textile	Pesticides, nitrate, nitrite, dyes
13	Yellow	Ultrasound, hydrodynamic cavitation, Fenton, photo-Fenton, TiO_2_	Pharmaceutical	Paracetamol, phenol,
14	Purple	Fenton, sonolysis, ultrasonic radiation	Papermaking	Herbicides, organic pollutant, COD
15	Light Blue	Solar irradiation	Drinking, tannery	COD, dyes
16	Orange	Ozonation	Drinking, tannery	Phenol, COD
